# IPCARF: improving lncRNA-disease association prediction using incremental principal component analysis feature selection and a random forest classifier

**DOI:** 10.1186/s12859-021-04104-9

**Published:** 2021-04-01

**Authors:** Rong Zhu, Yong Wang, Jin-Xing Liu, Ling-Yun Dai

**Affiliations:** 1 School of Computer Science, Qufu Normal University, Rizhao, China; 2 Department of Internet of Things Engineering, Wuxi Taihu University, Wuxi, China; 3 Experimental Teaching Center, Qufu Normal University, Rizhao, China

**Keywords:** LncRNA-disease, Association prediction, Incremental principal component analysis, Random forests

## Abstract

**Background:**

Identifying lncRNA-disease associations not only helps to better comprehend the underlying mechanisms of various human diseases at the lncRNA level but also speeds up the identification of potential biomarkers for disease diagnoses, treatments, prognoses, and drug response predictions. However, as the amount of archived biological data continues to grow, it has become increasingly difficult to detect potential human lncRNA-disease associations from these enormous biological datasets using traditional biological experimental methods. Consequently, developing new and effective computational methods to predict potential human lncRNA diseases is essential.

**Results:**

Using a combination of incremental principal component analysis (IPCA) and random forest (RF) algorithms and by integrating multiple similarity matrices, we propose a new algorithm (IPCARF) based on integrated machine learning technology for predicting lncRNA-disease associations. First, we used two different models to compute a semantic similarity matrix of diseases from a directed acyclic graph of diseases. Second, a characteristic vector for each lncRNA-disease pair is obtained by integrating disease similarity, lncRNA similarity, and Gaussian nuclear similarity. Then, the best feature subspace is obtained by applying IPCA to decrease the dimension of the original feature set. Finally, we train an RF model to predict potential lncRNA-disease associations. The experimental results show that the IPCARF algorithm effectively improves the AUC metric when predicting potential lncRNA-disease associations. Before the parameter optimization procedure, the AUC value predicted by the IPCARF algorithm under 10-fold cross-validation reached 0.8529; after selecting the optimal parameters using the grid search algorithm, the predicted AUC of the IPCARF algorithm reached 0.8611.

**Conclusions:**

We compared IPCARF with the existing LRLSLDA, LRLSLDA-LNCSIM, TPGLDA, NPCMF, and ncPred prediction methods, which have shown excellent performance in predicting lncRNA-disease associations. The compared results of 10-fold cross-validation procedures show that the predictions of the IPCARF method are better than those of the other compared methods.

**Supplementary Information:**

The online version contains supplementary material available at 10.1186/s12859-021-04104-9.

## Background

Bioinformatics has received increasing attention from both the public and the scientific community as biomedicine and sequencing technology developed. In bioinformatics, regions of the human genome that do not encode protein sequences are usually transcribed as noncoding RNAs (ncRNAs) [1]. Based on the length of such transcripts, ncRNAs can be partitioned into small ncRNAs and long ncRNAs (lncRNAs). The difference is that lncRNAs are more than 200 nucleotides in length [2], and they comprise the vast majority of noncoding RNAs. In recent years, lncRNAs have attracted wide attention from researchers. Increasing evidence indicates that lncRNAs usually play carcinogenic or tumour suppressor roles in human cancers [3, 4], including prostate cancer [5], hepatocellular carcinoma (HCC) [6] , colon cancer [7] , lung cancer [8], bladder cancer [9], and others.

lncRNAs have attracted wide attention from researchers in recent years. However, many lncRNA characteristics are still unclear, including their transcriptional regulation, structures, various biological processes or functions, and the molecular mechanisms of various diseases. At present, some new lncRNAs are discovered every year. This increasing number of lncRNAs has made using biological experimental methods for identifying lncRNA-disease associations more challenging. The use of biological experiments to identify lncRNA-disease associations introduces bottlenecks due to their experimental time and cost requirements. Thus, predicting potential lncRNA-disease associations through computational methods could effectively reduce the screening range of biological experiments, thereby also reducing the time and cost of biological experiments. In addition, using predictive calculation methods will help to discover the causes and mechanisms of diseases as soon as possible, which is highly important in disease diagnosis, drug prognosis, and target discovery.

As this research field has deepened, several lncRNA-disease association databases have been compiled. The LncRNADisease [10] is an lncRNA-disease association database established in 2013, and it was the first database in this area. Lnc2Cancer [11] was established in 2015; this dataset mainly includes data associations between cancer and lncRNAs. Compared with LncRNADisease, the entries in Lnc2Cancer are more comprehensive and complete. NONCODE [12] is a comprehensive knowledge base containing almost all ncRNAs, and LNCipedia [13] is a comprehensive human lncRNA database. By integrating a variety of data, the current version contains 120,353 human lncRNA transcripts. Moreover, it provides a tool for predicting protein-coding capabilities.

A semisupervised learning scheme called Laplacian regularized least squares for lncRNA-disease association (LRLSLDA) was proposed by Chen et al. [14] to predict new human lncRNA-disease associations. This was the first study to automatically predict lncRNA-disease associations. Later, Chen Xing made some improvements based on the LRLSLDA model. Sun et al. [15] proposed a global network-based computing framework (RWRlncD), in which a potential lncRNA-disease association is predicted by executing a random walk with restart (RWR) method on the lncRNA functional similarity network. A method for predicting potential lncRNA-disease associations by constructing lncRNA-disease association networks and rnRNA-disease bipartite networks was proposed by Yang et al. [16]. In 2015, a new hypergeometric distribution model (HGLDA) was developed by Chen et al. [1] to predict potential lncRNA-disease associations. Zhou et al. [17] proposed the RWRHLD method, which integrated the miRNA-related lncRNA-lncRNA crosstalk network, the disease similarity network, and the known lncRNA-disease-related network into a new network and then predicted potential lncRNA-disease associations based on the integrated network.

The above prediction models provide different perspectives and research ideas for the predicting lncRNA-disease associations and usher in the beginning of lncRNA-disease prediction. These methods provided reference data for the study of disease mechanisms and the functions of lncRNAs. However, the existing models still have some shortcomings; they are complex, suffer from high computational complexity, and neglect parameter selection. Therefore, considerable research on lncRNA-disease association prediction remains to be conducted.

The existing methods for predicting the lncRNA-disease associations have achieved solid results, but they have some limitations, and much room still exists for improvement. In this study, we develop a new automated method for predicting lncRNA-disease associations based on incremental principal component analysis (IPCA) and random forest (RF) technology, which we named IPCARF. First, we integrated disease semantic similarity, lncRNA functional similarity, and Gaussian interaction spectrum kernel similarity to obtain characteristic vectors of lncRNA-disease pairs. Second, we apply the IPCA method to effectively reduce the feature dimension of the dataset and obtain the best feature subspace from the original feature set. Finally, we train an RF model to predict potential lncRNA-disease associations.

## Results

All the experiments were done in the Python 3.7 software on the Keras library with a TensorFlow background.

### Selecting a classification algorithm

To choose an optimal classifier, we first compared the prediction results of several classic classifier algorithms on the experimental dataset. We compared RF classifiers with logistic regression (LR), k-nearest neighbor (KNN), linear discriminant analysis (LDA), naive bayes (NB), and support vector machine (SVM) algorithms. The parameters of all the algorithms were temporarily used as default parameter values.

**Fig. 1:**
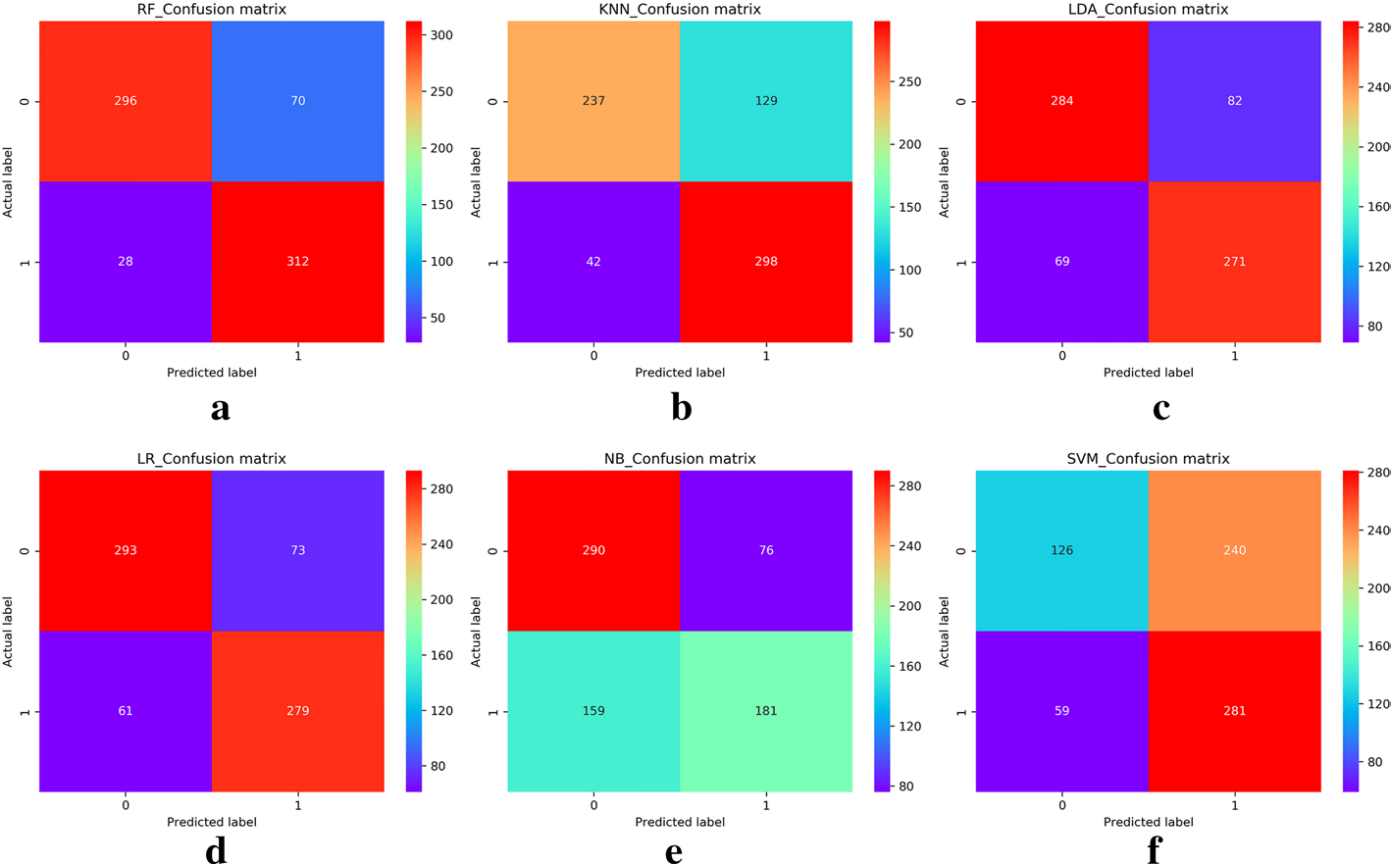
Confusion matrix of six algorithms (this figure is generated in the Python language environment with a 3.7 version)

First, we calculated and visualized confusion matrices based on the results of the six types of algorithms. The results are shown in Fig. 1. The horizontal axes of these confusion matrices denote the predicted label values, and the vertical axes denote the true label values. The dark colour on the diagonals indicates the classification accuracy. The darker the colour, the higher the accuracy. Figure 1 intuitively shows that among the six classification algorithms, the RF algorithm obtains the best results.

**Fig. 2:**
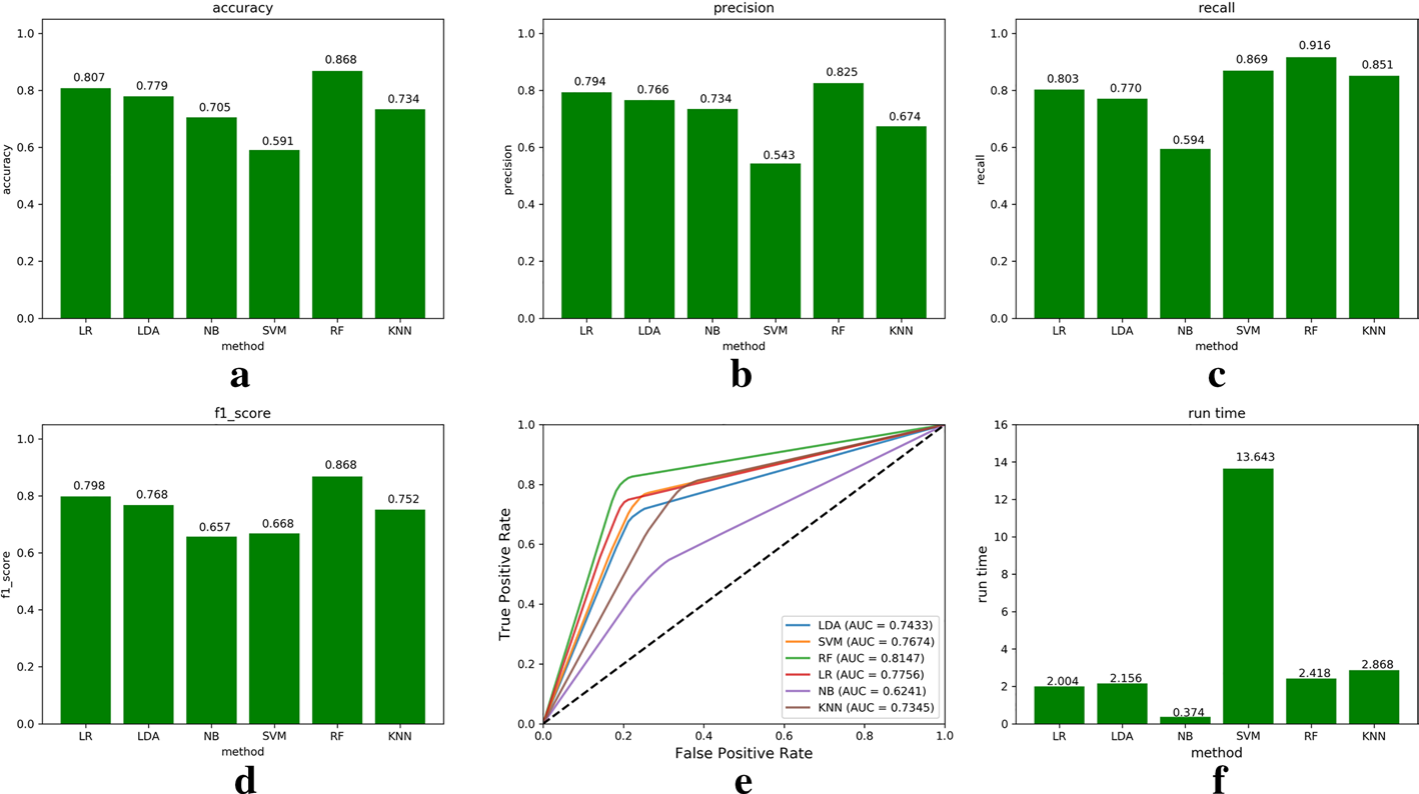
Evaluation results of six algorithms (this figure is generated in the Python language environment with a 3.7 version)

Cross-validation is a commonly used method in machine learning that can greatly reduce errors caused by sample selection. In our experiments, we used 10-fold cross-validation (10CV) to assess the classification prediction ability of six different classification algorithms. The detailed results of these six different methods are shown in Fig. 2, where (a)–(e) show that the accuracy, precision, recall, F1-score, and AUC values predicted by the RF algorithm are 0.868, 0.825, 0.916, 0.868, and 0.8147, respectively. Among the six algorithms, the RF algorithm achieves the highest values on all five evaluation indicators.

**Fig. 3:**
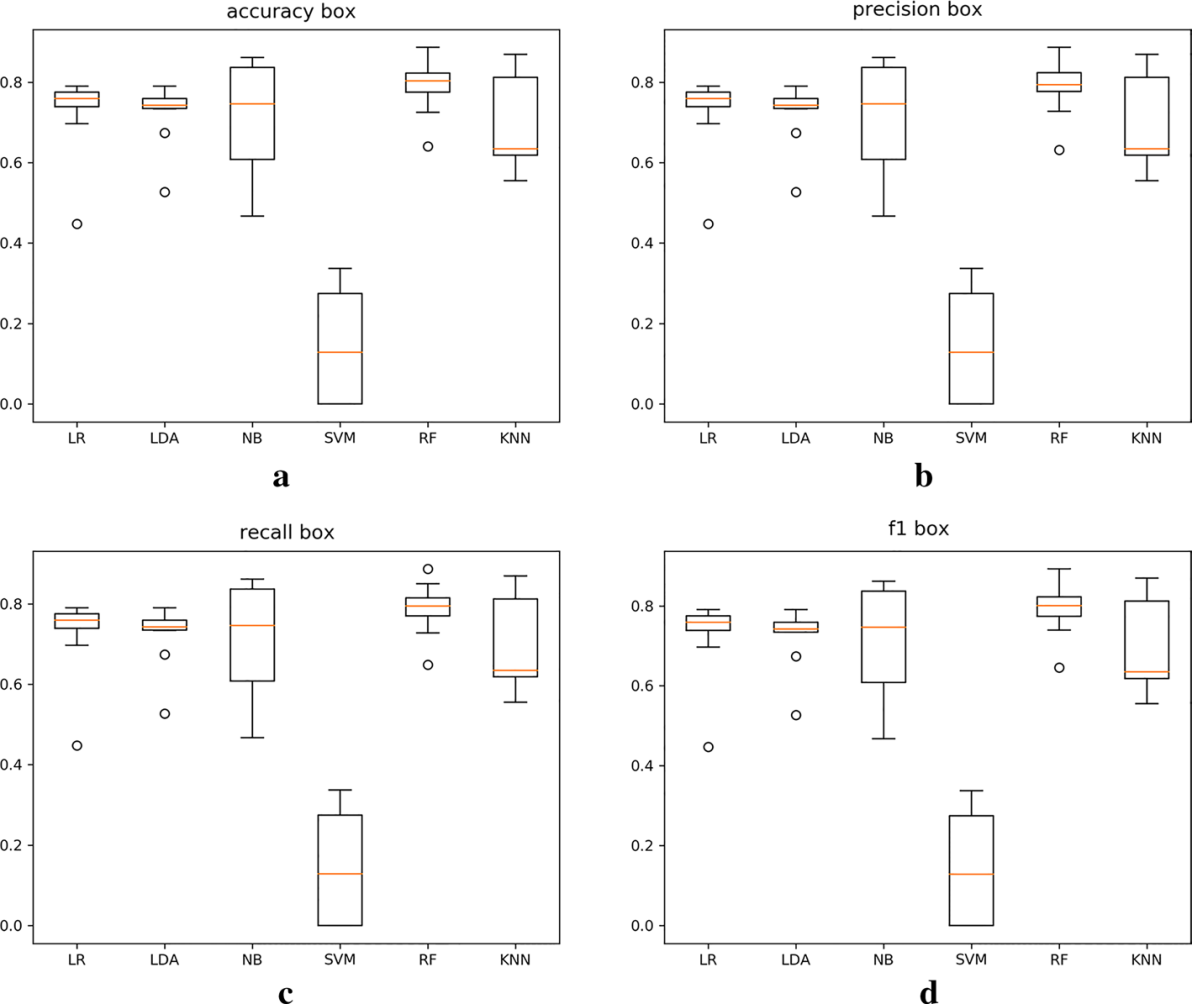
Box plots of evaluation results of six algorithms (this figure is generated in the Python language environment with a 3.7 version)

In the experiment, we also recorded the execution times of the six algorithms to compare and evaluate the runtime efficiency of each algorithm. The runtime comparison chart for the six algorithms in Fig. 2f shows that the SVM algorithm has the longest execution time, while the NB algorithm has the shortest, but the prediction results of these algorithms (such as the accuracy of the NB algorithm) are not as good as those of the RF algorithm. Therefore, we choose the RF algorithm as the experimental classifier. Figure 3 shows a box plot of the prediction results of the six classifiers using 10CV.

The results are further verified based on the experimental results shown in Fig. 3. The RF algorithm obtains the best prediction results among the six algorithms. Therefore, in our model, we chose the RF algorithm to integrate with the IPCA method to predict lncRNA-disease associations.

### Comparison of the proposed IPCARF and the traditional RF algorithm

**Fig. 4:**
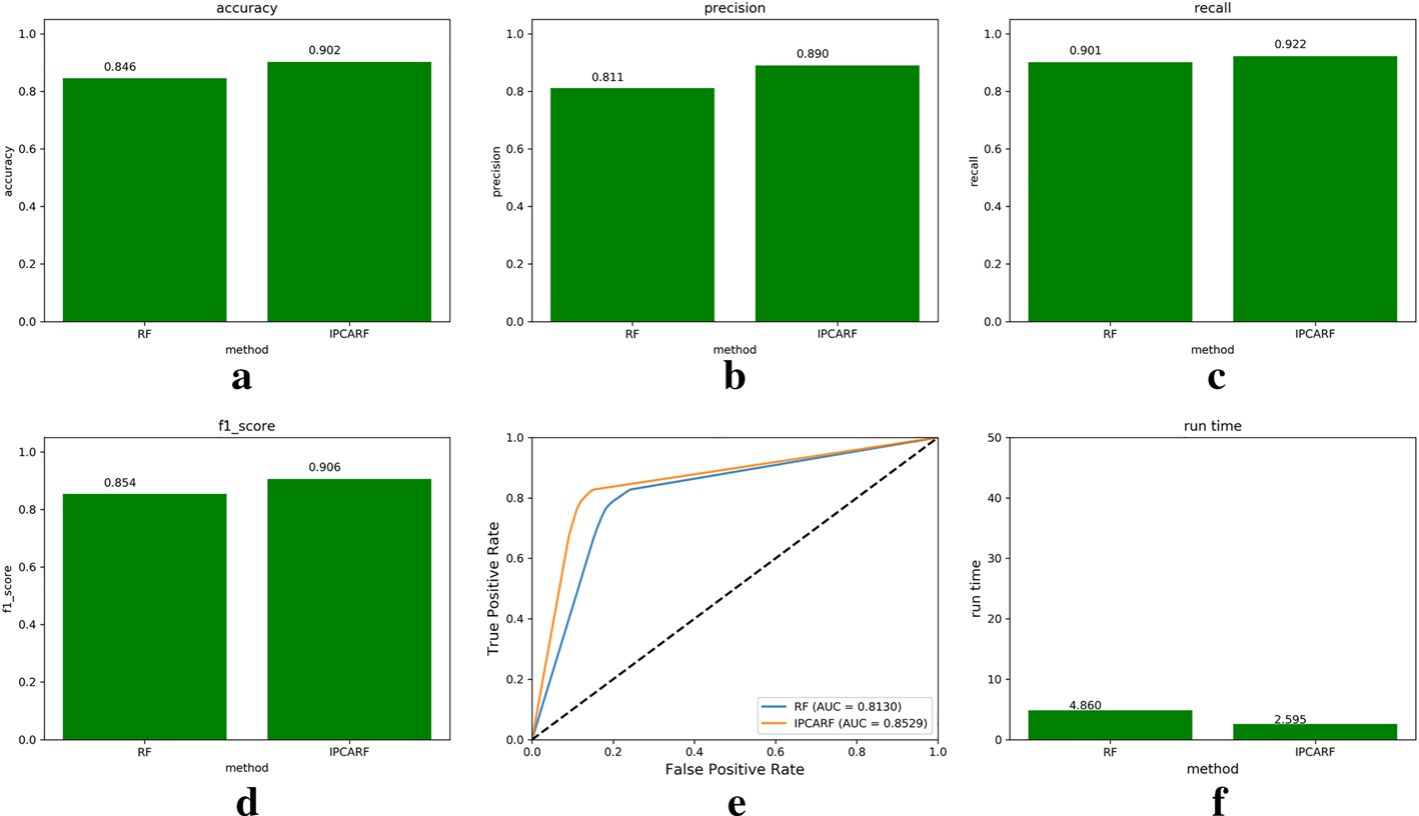
Comparison of prediction results of IPCARF and RF algorithms (this figure is generated in the Python language environment with a 3.7 version)

Through the above experiment, we selected the RF classifier as the classifier in the improved algorithm. Next, we used the IPCA algorithm to improve the performance of the RF classifier. We compared the prediction effect of the IPCARF algorithm with that of the traditional RF algorithm using 10CV. The experimental results are shown in Fig. 4, which shows that the accuracy, precision, recall, and F1-score values obtained when using the IPCARF algorithm for prediction are higher than those obtained when using only the RF algorithm for prediction. The ROC curve results verify that the prediction result of the IPCARF method is better than that of the RF method. In addition, the runtime of the IPCARF algorithm is lower than that of the RF. Therefore, it can be concluded that introducing the IPCA algorithm into the RF model effectively improves the performance of the classifier.

## Discussion

### Analysis of parameters

In the above experiment, we did not consider the effect of different parameter values on the prediction results of the algorithms; we used the default parameter settings for all the algorithms. In practical applications, after selecting a suitable model, the parameter settings are particularly important Because different parameters have different effects on model predictive ability.

The parameters used in IPCARF also affect its prediction performance. We have performed many experiments and found that, except for the n_estimators parameter, changes in the other parameters have relatively little impact on the prediction results of IPCARF. Therefore, here, we consider only the influence of the n_estimator parameter on the prediction results of the IPCARF algorithm. In this experiment, we set the value range of the n_estimators parameter to [100, 500, 1000, 1500, 2000, and 2500]; then, we selected the optimal n_estimator parameter value using the grid search (GS) method.

The grid search method is a commonly used parameter optimization algorithm [18]. A grid search is a method of finding parameters. Its core principle is to first define the parameter area to be searched and then divide the area into grids. The intersections in the grid form the parameter combinations to be searched. In other words, all the intersections in the grid are parameter combinations (c, g) that should be searched, and each combination (c, g) is retrieved during the grid search process. To obtain the best (c, g) combination, the k-fold method is used to test the classification accuracy of each group (c, g), and the group with the highest accuracy among all selected (c, g) is selected as the parameters for building the model.

**Fig. 5:**
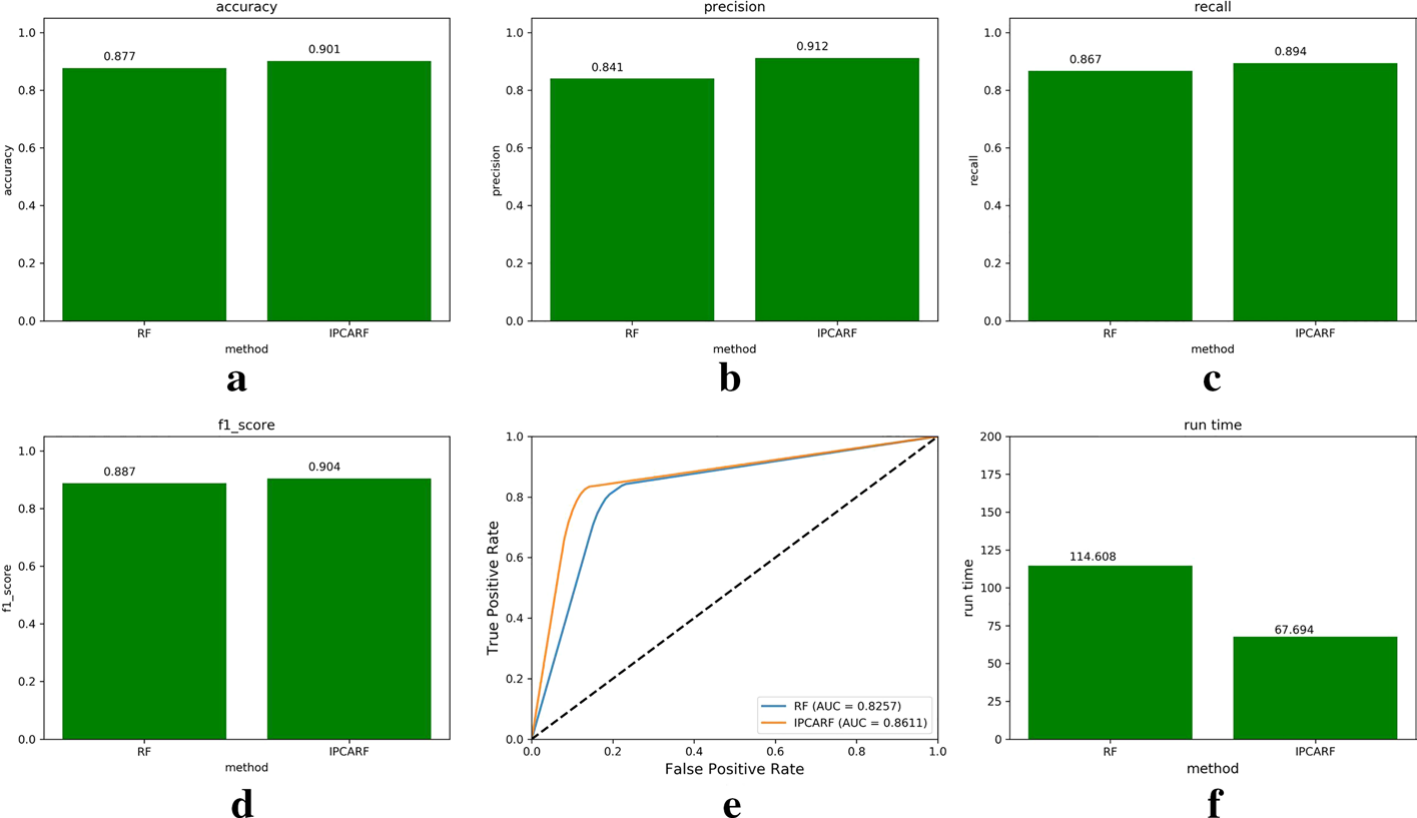
Comparison of prediction results of IPCARF and RF algorithms(n_estimators = 1500) (this figure is generated in the Python language environment with a 3.7 version)

In our experiment, the best n_estimator parameter value found after executing the GS algorithm was 1500. Thus, we adopt n_estimators = 1500 to further compare the execution performances of the IPCARF and RF algorithms. The experimental results are shown in Fig. 5.

Figure 5a–d displays the accuracy, precision, recall, and F1-score results of the two algorithms. Figure 5e shows that the AUC values predicted by the two algorithms are 0.8257 and 0.8611, and Fig. 5f shows that the running time of the IPCAFR algorithm is significantly lower than that of the RF algorithm.

### Comparisons with existing works

**Fig. 6:**
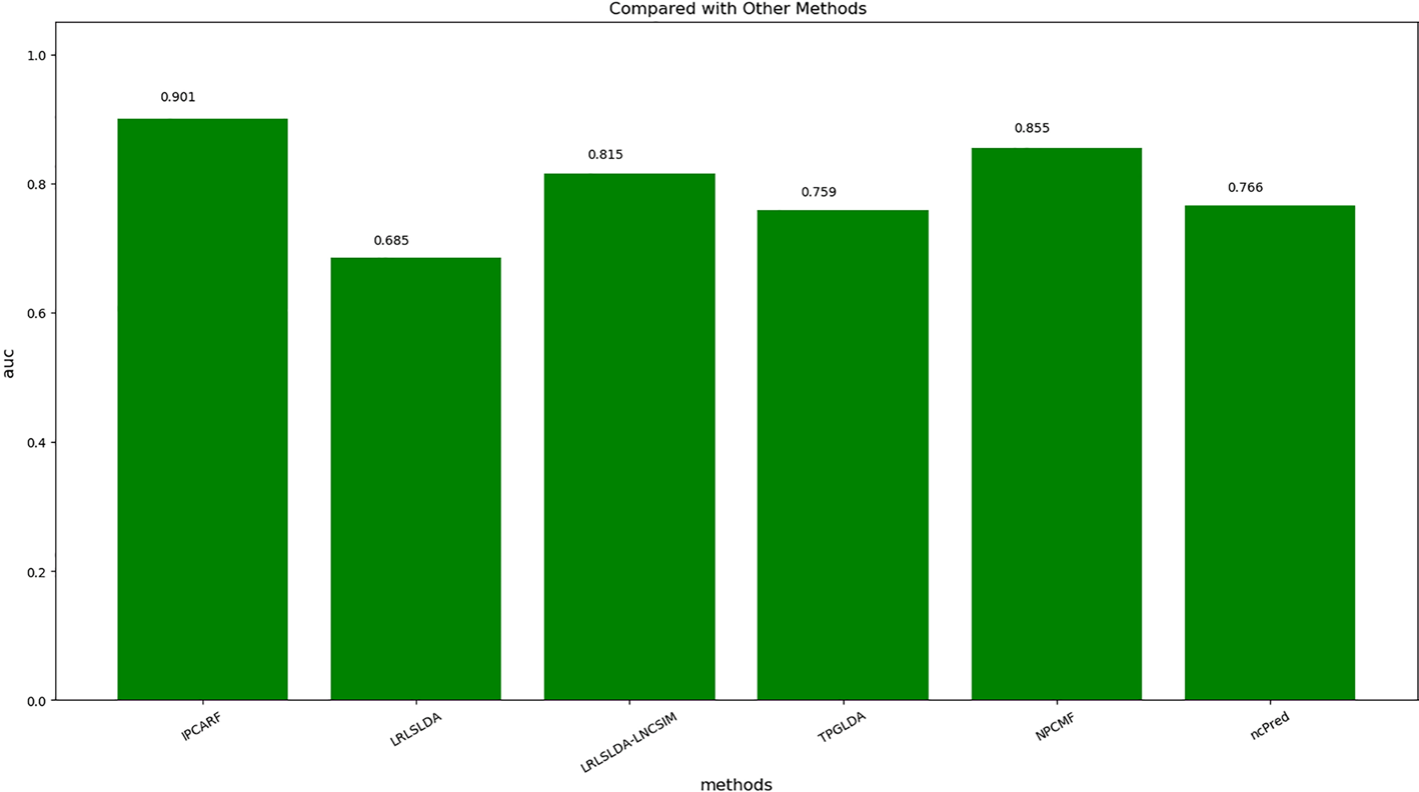
Comparisons with existing work (this figure is generated in the Python language environment with a 3.7 version)

Previous scholars have developed many effective prediction methods for the prediction of lncRNA-disease associations. However, because the data themselves have problems such as instability and because the evaluation methods used by various methods are inconsistent, the current methods still leave considerable room for improvement. To further verify the effect of IPCARF, we compared it with five other existing works, including LRLSLDA [14], LRLSLDA-LNCSIM [1], TPGLDA [19], NPCMF [20], and ncPred [21].The comparison results showing the AUC values of these algorithms are shown in Fig. 6.

Figure 6 shows that the AUC value obtained when using the IPCARF method to predict lncRNA-disease associations is better than that of the other comparison algorithms. Because of the instability of genetic data, the results of each experimental run differ to some degree. Consequently, we repeated the experiment 10 times and took the average as the final result. In the experiment, the highest value of AUC obtained when running the IPCARF algorithm was 0.906, and the lowest value was 0.861. These experimental results indicate that the prediction performance of the IPCARF method is slightly better than that of the comparative methods.

### Case study

Lung cancer is a common malignant lung tumor. The top 5 long non-coding RNAs that use the IPCARF algorithm to predict lung cancer are: GAS5,XIST,CDKN2B-AS1, PVT1 and HOTAIR. Four of the top 5 have the latest literature to verify. Ranked No. 1 is GAS5, and the research in the literature [22] shows that GAS5 may play a role in suppressing cancer. Ranked No. 2 is XIST, and the research in the literature [23] shows that XIST plays an important regulatory role in cancer biology. Ranked No. 4 is PVT1, and the research in the literature [24] shows that PVT1 can inhibit cell proliferation, migration and invasion. Ranked No. 5 is HOTAIR, and the literature [25] found that HOTAIR affects the drug resistance of small cell lung cancer cells by regulating the methylation of HOXA1.

## Conclusions

Biological experiments have continuously been the primary means of identifying lncRNA-disease associations. However, the number of newly discovered lncRNAs increases every year, and this growing amount of data functions as a bottleneck to the experimental identification methods. Fortunately, several publicly available databases have emerged that have introduced greater opportunities for predicting potential lncRNA-disease associations through computational methods. Using computational methods to predict potential lncRNA-disease associations is important, because such associations can effectively improve our understanding of disease pathogenesis and guide treatments. In this study, we proposed a novel model called IPCARF to predict lncRNA-disease associations and compared it with the existing LRLSLDA, LRLSLDA-LNCSIM, TPGLDA, NPCMF, and ncPred prediction methods using 10CV. These methods have achieved excellent performances for predicting lncRNA-disease associations. The comparison results show that the prediction results of the IPCARF method are better than those of the compared methods.

Although the IPCARF method has achieved good prediction results, it still has some limitations that should be improved in future studies. First, the experimental data are still not rich enough, which limits the predicted results. As more data related to lncRNA diseases becomes available, the IPCARF method will improve. The complexity and inconsistency of biological data also cause certain difficulties in improving and comparing algorithms, especially the inability to obtain completely consistent data sources. In future work, we will consider integrating data from different sources to improve the prediction performance of IPCARF by improving the integrity and quality of the experimental data.

## Methods

### Data collection

#### Disease similarity data

The data on disease similarity compiled by different scientific researchers are not the same. Among them, the data compiled by van Driel et al. [26] is the most often cited; it is also the most recognized and is considered to be relatively authoritative disease similarity data. A similarity network of 5080 human genetic diseases is constructed by this database, which is available at http://www.cmbi.ru.nl/MimMiner/. The database has a matrix file format.

#### lncRNA-disease association data

In 2013, Chen et al. [10] established the LncRNADisease database (http://210.73.221.6/lncrnadisease), which was the first database of lncRNA-disease association data, and it was manually collected and experimentally verified. Over time and the continuous expansion of lncRNA research, the LncRNADisease database has also continuously expanded, and the number of entries increases yearly. In this study, we used the v2017 data from the LncRNADisease database. The datasets generated and analyzed during the current study are presented in Additional file 1.

### Disease semantic similarity

#### Disease semantic similarity model

Referring to the calculation method in [1], two models are used on the directed acyclic graph (DAG) of diseases to compute a disease semantic similarity score.

First, the contribution of the disease term t in DAG(D) to the semantic value of disease D is defined as follows:

1 $$\begin{aligned} \left\{ \begin{matrix} C1_{A}(D)=1\\ C1_{A}(i)=max\left\{ \Delta *C1_{A}({i}')|{i}'\in children~~of~~i \right\} ~~if~~i\ne D , \end{matrix}\right. \end{aligned}$$

where $\Delta$ represents a semantic contribution attenuation factor.

Then, all the contributions of the ancestral disease and disease *D* itself are summed, and the semantic value of disease *D* is defined as follows:

2 $$\begin{aligned} G(D)=\sum _{i\in Disease(D)}C1_{D}(i). \end{aligned}$$

The semantic similarity between two diseases $D_1$ and $D_2$ is defined as follows:

3 $$\begin{aligned} sim1(D_1,D_2)=\frac{\sum _{i\in Disease(D_1)\cap Disease(D_2)}(C1_{D_1}(i)+C1_{D_2}(i))}{C1(D_1)+C1(D_2)}, \end{aligned}$$

where *sim*1 denotes the disease semantic similarity matrix.

Moreover, the method for calculating disease similarity refers to the calculation method proposed in [27], which provides a detailed description.

#### Gaussian interaction profile kernel similarity for disease

Similar diseases may have similar related lncRNAs. The similarity of Gaussian interaction kernels can be computed from the known lncRNA-disease association network. The Gaussian interaction kernel similarity between diseases *D*1 and *D*2 is computed as follows:

4 $$\begin{aligned} GKS(D_1,D_2)=exp(-k_{dis}\left\| D_1-D_2 \right\| ^{2}), \end{aligned}$$

where $-k_{dis}$ represents the standardized core width, which is calculated by

5 $$\begin{aligned} k_{dis}=\frac{1}{\frac{1}{m}\sum _{i=1}^m \left\| D(i) \right\| ^{2}}, \end{aligned}$$

where *m* represents the disease number.

### Gaussian interaction profile kernel similarity for lncRNA

The Gaussian interaction kernel similarity between lncRNAs $L_1$ and $L_2$ is computed as

6 $$\begin{aligned} GKS(L_1,L_2)&= exp(-k_{lnc}\left\| L_1-L_2 \right\| ^{2}), \end{aligned}$$

7 $$\begin{aligned} k_{lnc}= & {} \frac{1}{\frac{1}{n}\sum _{i=1}^n \left\| L(i) \right\| ^{2}} , \end{aligned}$$

where *n* represents the lncRNA number.

### The IPCA algorithm

#### The PCA algorithm

Principal Component Analysis (PCA) is a commonly used data analysis algorithm and an unsupervised linear feature extraction algorithm. PCA has been widely used in applications such as lossy data compression, feature selection, and dimensionality reduction [28]. PCA methods can reduce data from a high-dimensional space to a low-dimensional space because it merges similar features due to the variance. Thus, PCA can reduce both data and the number of data features, which helps to prevent model overfitting.

The main idea underlying the PCA algorithm is to describe things using fewer data features that represent most of the main information. PCA is a statistical method that recombines characteristic variables with linear associations into fewer characteristic variables. The PCA algorithm is essentially a transformation of the variables that introduces a set of new variables that are not related to the original variables; instead, these new variables are linear functions of the original variables. Each new variable is called a principal component. This group of principle is sorted based on variance; the first principal component is the one with the largest variance in the linear function. The second principal component is the linear function with the second-largest variance, and the first and second principal components are not correlated with each other. The third principal component is also uncorrelated with the first and second principal components and constitutes the linear function with the third-largest variance. By analogy, the original data are transformed using $K-L$ to obtain new data after dimensionality reduction.

Assume that the size of the original data sample matrix is $m\times n$. The matrix has *m* dimensions, and each dimension has *n* samples. The sample matrix [29] can be expressed as follows:

8 $$\begin{aligned} D=\begin{bmatrix} d_{11} &{} \quad d_{12} &{} \quad \cdots &{}\quad \quad d_{1n}\\ d_{21} &{} \quad d_{22} &{}\quad \cdots &{}\quad d_{2n} \\ \cdots &{}\quad \cdots &{}\quad \cdots &{}\quad \cdots \\ d_{m1}&{} \quad d_{m2} &{} \quad \cdots &{} \quad d_{mn} \end{bmatrix} \end{aligned}$$

Find the zero-average of each row in the matrix *D* , that is, subtract the average value of each column, expressed as follows:

9 $$\begin{aligned} D=\begin{bmatrix} d_{11}-a_1 &{} \quad d_{12}-a_2 &{} \quad \cdots &{} \quad d_{1n}-a_n\\ d_{21}-a_1 &{} \quad d_{22}-a_2 &{}\quad \cdots &{}\quad d_{2n}-a_n \\ \cdots &{}\quad \cdots &{}\quad \cdots &{} \quad \cdots \\ d_{m1}-a_1&{} \quad d_{m2} -a_2&{} \quad \cdots &{} \quad d_{mn}-a_n \end{bmatrix} \end{aligned}$$

where $a_{i}$ represents the average of each column of samples, expressed as follows:

10 $$\begin{aligned} a_{i}=\frac{1}{m}\sum _{i=1}^{m}d_{ji}. \end{aligned}$$

Then, calculate the covariance matrix of the sample matrix. For an $m\times n$ sample matrix, the covariance matrix *C* is an $m\times m$ matrix, and each element $C_{ij}$ of the covariance matrix represents the covariance of the variable $d_{i},d_{j}$.

Next, compute the eigenvalues of the covariance matrix and sort the calculated eigenvalues in descending order. The eigenvectors relevant to the first k eigenvalues are adopted to form a new matrix.

Finally, the projection of the original data sample matrix *D* on the new eigenvector matrix is calculated to obtain the data eigenvectors after dimensionality reduction.

#### The IPCA algorithm

The IPCA algorithm mainly improves the covariance matrix and reconstructs the original covariance matrix into a low-dimensional matrix that retains most of the information of the original covariance matrix.

First, the $l_{2}\text {-}norm$ of each column vector of the original covariance matrix is calculated as follows:

11 $$\begin{aligned} \left\| b_{j} \right\| _{2}=\sqrt{\sum _{i=1}^{m}\left| c_{ji} \right| ^{2}} . \end{aligned}$$

Next, form a new matrix *B* with the largest top *k* column vectors in the obtained norm.

Perform *QR* decomposition on the new matrix *B* to obtain the low-dimensional matrix *C*1.

Perform singular value decomposition on the *C*1 matrix. Arrange the obtained singular value representations in order of importance, discard the unimportant eigenvectors, and retain the eigenvalues of the data set after dimensionality reduction.

In short, in the IPCA algorithm, the singular value decomposition of the central data is used for linear dimensionality reduction, and only the most important singular vectors are retained to project the data into a lower-dimensional space.

#### The RF classification algorithm

The RF classification algorithm belongs to the supervised learning subfield of the machine learning field. It uses samples from a dataset for training and the trained model is applied to perform predictions on real data to evaluate whether the results meet expectations.

The traditional classification algorithms mainly include k-nearest neighbour (KNN) [30] algorithms, naive Bayes (NB) [31] algorithms, decision tree algorithms and support vector machine (SVM) [32] algorithms. Most of these algorithms are relatively mature, and each has a range of suitable application scenarios, but they also leave space for corresponding algorithm classification performance improvements. The decision tree algorithm is a type of split tree approach based on data attribute characteristics. As research has deepened, improved decision trees such as ID3, C4.5, classification and regression tree (CART), and regression trees have gradually been developed. The decision tree algorithm has advantages such as an easy way to understand the decision results and powerful functions, but it may exhibit problems such as weak fitting. The NB algorithm comes from the field of statistics and predicts the posterior probability based on the prior probability. The advantage of the NB algorithm is its fast calculation speed, while its disadvantage is that there may be dependencies between attributes, which often leads to lower classification accuracy. The SVM algorithm performs high-dimensional and nonlinear classification by constructing a hyperplane. The advantages of the SVM algorithm are that it is highly efficient and provides good classification accuracy. Its disadvantages are the complex structure of its kernel function and a lack of data sensitivity.

**Fig. 7:**
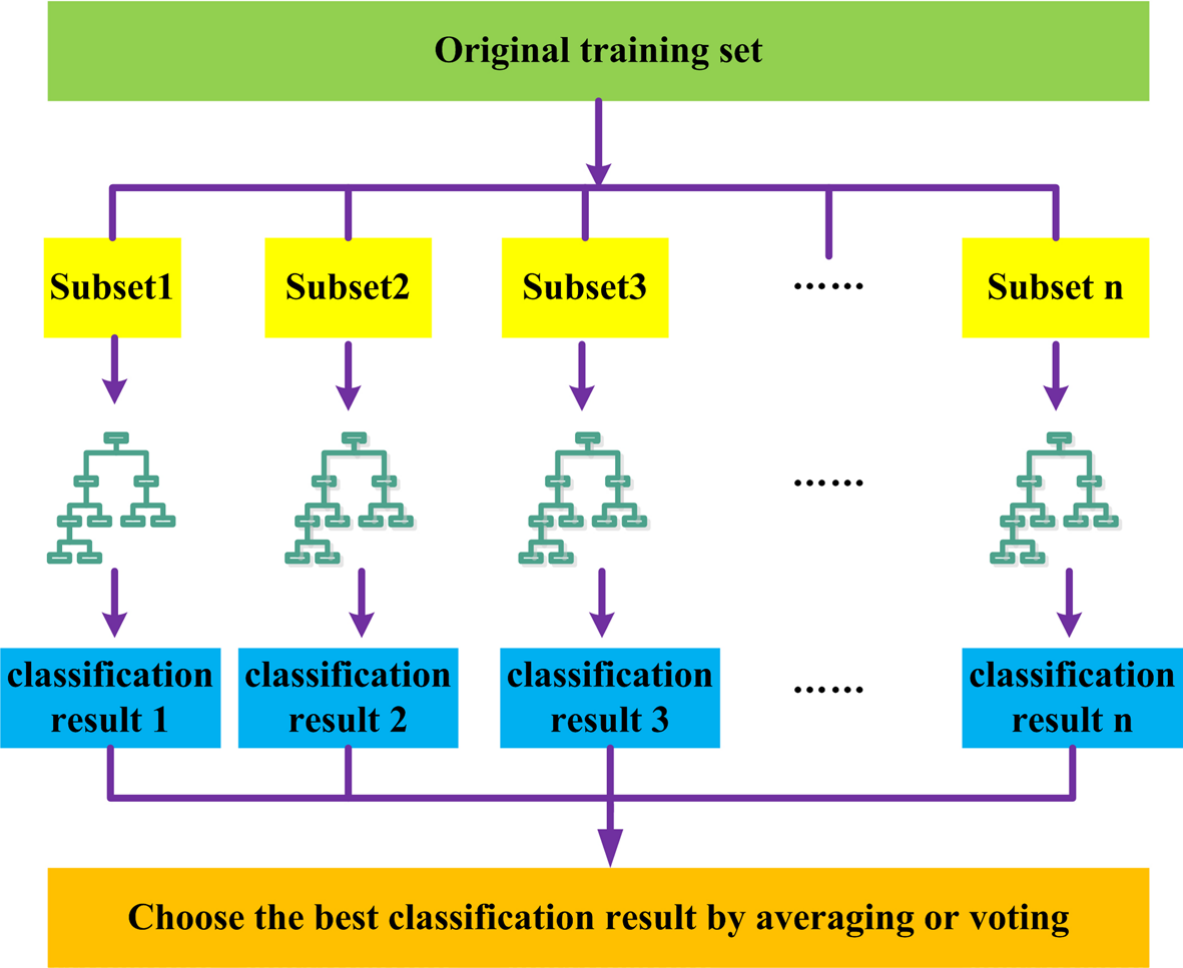
Flowchart of the rf (this figure is drawn manually using visio2010 software)

The RF method, first proposed by Breiman [33]is a machine learning algorithm consisting of many decision trees. It is a combination of the Bagging [34] and Random Subspaces [35]methods. The RF algorithm [35] is considered to be an ensemble learning and supervised classification method. It first randomly establishes a forest composed of multiple unrelated decision trees; these multiple decision tree classifier models each learn and perform prediction separately. Then, the prediction results of the multiple decision tree classifier models are combined to obtain a final prediction result. There are two typical ways of combining the prediction results from different decision tree classifiers in RF. One is to average the prediction results of all the decision tree classifiers to obtain a prediction result for the entire forest. The other is to conduct a vote on the prediction results from all decision tree classifiers to select an optimal prediction result as the prediction result of the entire forest. A general flowchart of the RF algorithm is shown in Fig. 7.

The RF algorithm first selects n samples from the original training set as a training subset and then generates a decision tree for each subset. The above steps are repeated a total of n times to generate n decision trees that form the random forest. Finally, the random forest obtained by training is used to predict test samples, and an optimal classification result is selected using either the mean method or the voting method.

The hospital has a large amount of data after the diagnosis of the patient, how to extract the data that has a high correlation with the patient’s disease from this large and complex data set for analysis. If we can use some high-performance algorithms to efficiently classify these data and make predictions for some diseases, such as the predictive analysis of cancer and other diseases, it will have very important and far-reaching significance. The data processed in the medical field are usually high-dimensional, and many data sets are extremely unbalanced. Traditional analysis methods cannot get a good diagnosis effect. The random forest can efficiently process high-dimensional data, so it is widely used in the medical field.

#### Long noncoding RNA-disease prediction based on IPCA and RF

**Fig. 8:**
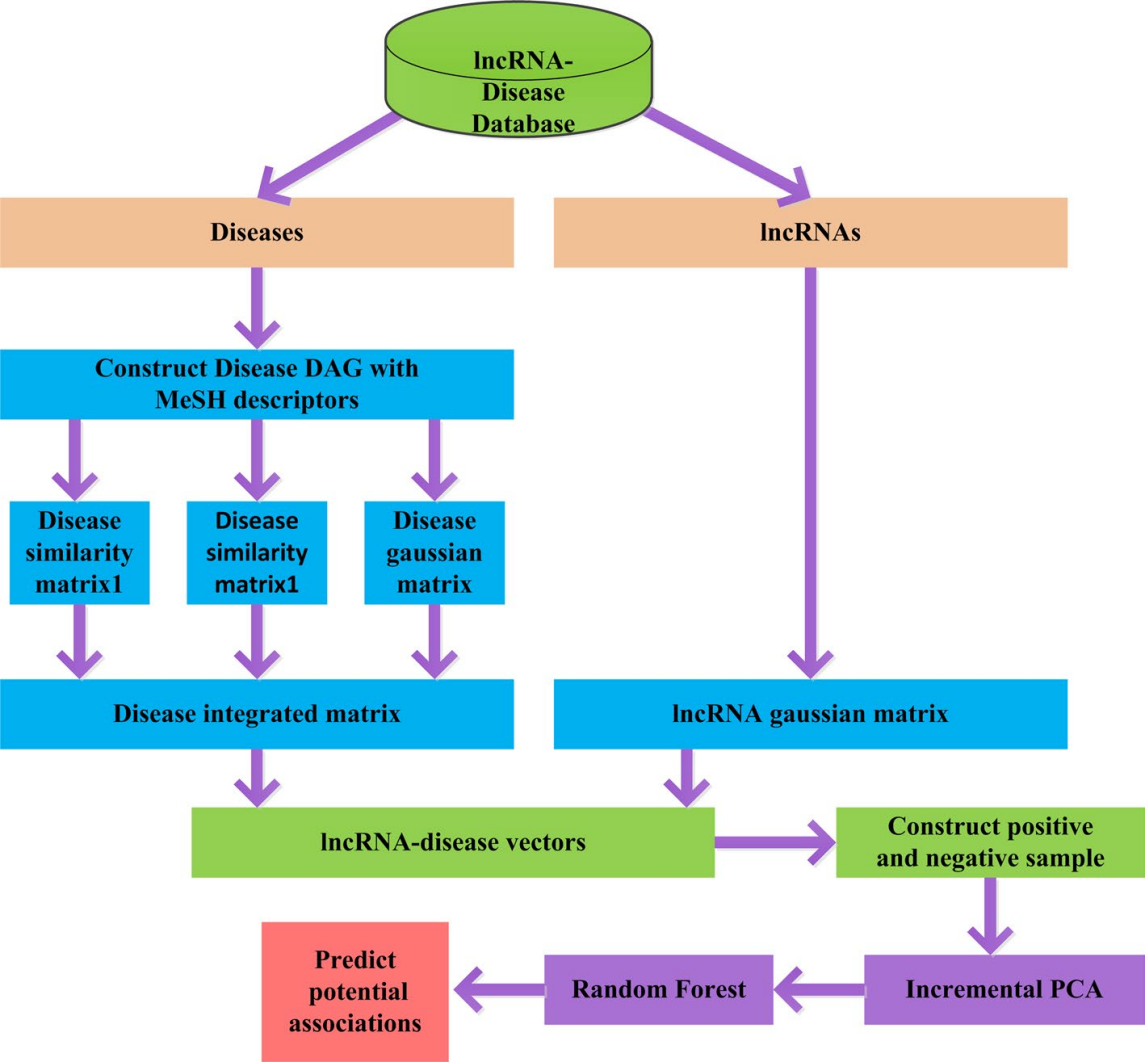
Flowchart of IPCARF (this figure is drawn manually using Visio2010 software)

In this study, we developed an algorithm called IPCARF based on the IPCA and RF methods. First, two semantic similarity matrices, a Gaussian kernel similarity matrix for diseases and a Gaussian kernel similarity matrix for lncRNAs are established. Second, a feature vector is extracted from the similarity matrix to construct an adjacency matrix. Then, the positive samples and negative samples are extracted from the adjacency matrix to construct the dataset for prediction. Next, the IPCA method is applied to select features and reduce the dataset dimensionality. Finally, the FR classifier is used to make predictions. The IPCARF process is shown in Fig. 8.

### Evaluation metrics

To assess the potential classification prediction ability of the IPCARF algorithm, we adopted the metrics of precision, accuracy, F1-score, recall, and the receiver operating characteristic (ROC) curve to represent the abilities of the six candidate algorithms. The calculation formulas for several of these metrics are defined below:

12 $$\begin{aligned} accuracy= & {} \frac{TP+TN}{TP+FN+FP+TN} , \end{aligned}$$

13 $$\begin{aligned} precision= & {} \frac{TP}{TP+FP} , \end{aligned}$$

14 $$\begin{aligned} recall= & {} \frac{TP}{TP+FN} , \end{aligned}$$

15 $$\begin{aligned} f1\text {-}score= & {} \frac{2 \times accuracy \times recall}{accuracy+recall} , \end{aligned}$$

where *TP* is the number of positive samples correctly classified as positive samples by the classifier; *TN* is the number of negative samples correctly classified as negative samples by the classifier; *FP* is the number of negative samples incorrectly classified as positive samples by the classifier; and *FN* is the number of positive samples incorrectly classified as negative samples by the classifier.

Recall is the proportion of positive examples that are accurately predicted (it can be called TPR or recall), that is, the proportion of positive examples correctly predicted by the classification model to the total number of correctly classified samples. The higher the accuracy, precision, recall, and F1-score are, the better the classification performance is.

The ROC curve is a characteristic of classifier performance. The abscissa of this curve is the false positive rate (FPR), and the ordinate is TPR (recall). The formula for calculating the FPR is shown below:

16 $$\begin{aligned} FPR=\frac{FP}{FP+TN} . \end{aligned}$$

The area under the curve (AUC) represents the area under the ROC curve enclosed by the coordinate axis. The value of this area cannot exceed 1. Usually, the ROC curves are located above the straight line $y=x$. Generally, the AUC value should range between 0.5 and 1. An AUC value closer to 1.0 represents a better classifier performance. An $AUC <= 0.5$ has no application value. Because the ROCs evaluate model results in an objective manner, this metric is widely used in practical applications.

## Supplementary Information

**Additional file 1**. LncRNA name, Disease name, Dysfunction type.

## Data Availability

This database is available at http://www.cuilab.cn/lncrnadisease. Source code is available at https://github.com/zhurong1942/IPCARF_zr1.

## Abbreviations

IPCA: Incremental principal component analysis
RF: Random forests
lncRNA: Long non-coding RNA
DAG: Directed acyclic graph
GS: Grid search
AUC: Area under curve
10CV: 10-Fold cross-validation
ROC: Receiver operating characteristic
LR: Logistic regression
KNN: K-nearest neighbor
NB: Naive Bayes
LDA: Linear discriminant analysis
SVM: Support vector machine
CART: Classification and regression tree
FN: False negative
FP: False positive
TN: True negative
TP: True positive
FPR: False positive rate
CART: Classification and regression tree
TCGA: The Cancer Genome Atlas
LRLSLDA: Laplacian Regularized Least Squares for LncRNA-Disease Association
LNCSIM: lncRNA functional similarity calculation models
TPGLDA: lncRNA-diseasegene tripartite graph
NPCMF: Nearest profile-based collaborative matrix factorization
ncPred: ncRNA-disease association prediction
